# The effectiveness of health care provider physical activity recommendations in cancer survivors: a systematic review and meta-analysis protocol

**DOI:** 10.1186/s13643-017-0453-3

**Published:** 2017-03-27

**Authors:** Jennifer Brunet, Amanda Wurz, Connor O’Rielly, Doris Howell, Mathieu Bélanger, Jonathan Sussman

**Affiliations:** 1 School of Human Kinetics, University of Ottawa, 125 University Private, Montpetit Hall, Room 339, Ottawa, Ontario K1N 6N5 Canada; 2 Institut de Recherche de l’Hôpital Montfort, l’Hôpital Montfort, Ottawa, Ontario Canada; 3 Cancer Therapeutic Program, Ottawa Hospital Research Institute, Ottawa, Ontario Canada; 4 Faculty of Nursing, University of Toronto, Toronto, Ontario Canada; 5 University Health Network, (Princess Margaret Cancer Centre), Toronto, Ontario Canada; 6 Department of Family Medicine, Université de Sherbrooke, Moncton, New Brunswick Canada; 7 Centre de Formation Médicale du Nouveau-Brunswick, Moncton, New Brunswick Canada; 8 Vitalité Health Network, Moncton, New Brunswick Canada; 9 Department of Oncology, McMaster University, Hamilton, Ontario Canada

**Keywords:** Systematic review, Physical activity, Behaviour change, Cancer, Health care providers

## Abstract

**Background:**

Cancer survivors face a range of negative physical and psychological effects that can be mitigated by participating in physical activity. Despite this, most do not meet recommended levels. Health care providers may be in a unique position to promote participation in physical activity among cancer survivors. The aim of this systematic review and meta-analysis is to synthesize the findings from randomized controlled trials and controlled clinical trials investigating the effectiveness of health care provider-administered physical activity recommendations on participation in physical activity among cancer survivors.

**Methods/design:**

Ten electronic databases (CINAHL, CENTRAL, Education Source, EMBASE, LILACS, MEDLINE, OTSeeker, PEDro, PsycINFO, SPORTDiscus) will be searched to identify relevant studies. The electronic searches will be supplemented by scanning the reference lists of relevant articles retrieved during these searches to ensure all potentially relevant studies are identified. Two reviewers will independently screen all titles and abstracts resulting from the searches to identify potentially eligible studies. They will then screen the full-text articles passing the first screen to identify studies for inclusion using predetermined inclusion/exclusion criteria, extract data from studies meeting all criteria, and assess the risk of bias of these studies. Results will be summarized narratively and statistically.

**Discussion:**

By summarizing the best available evidence for the effectiveness of health care provider physical activity recommendations for increasing participation in physical activity among cancer survivors, the results of this systematic review and meta-analysis will help determine if making physical activity recommendations effectively changes cancer survivors behaviour. It will also help to identify knowledge gaps and highlight areas in need of additional research.

**Electronic supplementary material:**

The online version of this article (doi:10.1186/s13643-017-0453-3) contains supplementary material, which is available to authorized users.

## Background

Among the 1.8 million adults who are expected to be diagnosed with cancer in North America [1, 2], over 63% are expected to live at least 5 years following their diagnosis [1, 2]. However, continued physical and psychological side effects (e.g. musculoskeletal symptoms, impaired cardiovascular system functioning, cancer-related fatigue, cognitive problems, anxiety, depression) are common [3–6]. These effects may result in functional limitations, delayed return to work, impaired performance/productivity, reduced physical and psychological health, and continued use of health care resources (e.g. [7–11]).

Various therapies are now available (e.g. pharmacotherapy, psychological therapies, behavioural therapies), where the goal is to relieve side effects, prolong life, and/or improve quality of life. Physical activity is one therapeutic option for cancer survivors as it can help to mitigate many of the negative side effects cancer survivors experience during and following treatment. The safety and effectiveness of physical activity for improving physical (e.g. strength, cardiovascular system functioning, body composition) and psychological (e.g. anxiety, stress, distress, depression, quality of life) aspects of health as well as survival have been established [12–18]. Despite this evidence, most cancer survivors are not sufficiently active to achieve health benefits. Recent estimates suggest that only 20 to 30% of cancer survivors accumulate the recommended 150 minutes of moderate-to-vigorous intensity physical activity per week [19] —a level known to yield health benefits [20]. These statistics, coupled with evidence that physical inactivity is a significant risk factor for persistent symptoms and side effects, susceptibility to chronic conditions, all-cause mortality, and disease recurrence in cancer survivors [12–18], underscore the need to promote participation in physical activity among cancer survivors.

Health care providers are well placed to recommend physical activity to their patients in order to mitigate cancer-related side effects and promote overall health [21]. Although there is evidence showing that patients of physicians who recommend physical activity are more likely to participate in physical activity [22–26], the majority of cancer survivors still do not receive advice to participate in physical activity from health care providers [27–30]. Several barriers to recommending physical activity during clinical visits have been reported by health care providers, including a perceived lack of time, believing that it is not part of their role, a lack of knowledge/training in physical activity counselling, a perceived lack of acceptance or willingness of patients to follow recommendations, and anticipated failure in changing patients’ behaviour [29–33]. The latter may reduce health care providers’ willingness to recommend physical activity because they may be wary about the effectiveness of doing so [32]. Thus, summarizing the evidence for the effectiveness of providing physical activity recommendations can be useful to help health care providers see the advantage of promoting physical activity, and in turn ensure physical activity becomes routinely promoted in practice.

Therefore, the purpose of this systematic review and meta-analysis is to summarize the effectiveness of health care provider physical activity recommendations (intervention) as compared to usual care (comparator) on participation in physical activity (outcome) in cancer survivors (population) to answer the following research question: “Compared to usual care (i.e. the care as usually received by patients in daily practice and no physical activity recommendation), what is the effect of health care provider physical activity recommendations on cancer survivors’ participation in physical activity?”

## Methods

This review protocol has been prepared and reported in accordance with the Preferred Reporting Items for Systematic Review and Meta-Analysis Protocols (PRISMA-P) statement [34, 35]. The PRISMA-P statement contains a checklist that ensures complete and transparent reporting. See Additional file 1 for the completed PRISMA-P checklist.

### Inclusion criteria

To be included in this systematic review and meta-analysis, studies will need to meet the following inclusion criteria: (1) published in a peer-reviewed scientific journal, (2) have full-text available in English, and (3) be an original study. In the case that information is incomplete, missing, or unclear, corresponding authors of the study will be contacted to obtain the information required. In addition, specific criteria related to the types of participants, interventions, studies, and outcomes are detailed below.

#### Types of participants

Studies with participants that included adult (18 years or older) cancer survivors, defined as an individual from the point of cancer diagnosis onward [36], will be included. Cancer survivors could have been at any stage along the disease trajectory (i.e. at diagnosis, currently undergoing treatment, completed treatment, years following treatment, end-of-life).

#### Types of interventions

For the purpose of this review, an intervention will be defined as any health care provider-administered recommendation (e.g. advice, verbal or written prescription, behavioural counselling, referral to physical activity services, or otherwise) delivered with the intent of promoting participation in physical activity. Only studies where the recommendation was delivered in person during a clinical visit will be included. The health care provider could have been any health care or allied health care provider (e.g. oncologist, nurse, physician, physiotherapist, kinesiologist) within the cancer survivors’ circle of care.

#### Types of studies

The types of studies that will be included in this review are randomized controlled trials and controlled clinical trials that examined the effect(s) of physical activity recommendations administered by health care providers on participation in physical activity among cancer survivors in comparison to a control group receiving usual care as provided in everyday practice (i.e. no physical activity recommendation). These study designs were specifically chosen as they provide the most robust evidence [37, 38]. However, if during the searches the number of randomized controlled trials and controlled clinical trials do not appear to be sufficient to answer the research question (i.e. no or very few meet eligibility criteria), other study designs which are likely to provide valid data on the outcome of interest will be considered. Any modifications to the inclusion/exclusion criteria pertaining to the study design will be noted as a protocol amendment.

#### Types of outcome measures

The primary outcome measure of interest in this review is participation in physical activity. To be included, studies must have included data on participants’ participation in physical activity at least twice: pre- and post-intervention. Secondary outcomes to be reported on in this review will include physical and psychological health outcomes.

### Exclusion criteria

Studies including an intervention targeting multiple health behaviours (e.g., physical activity and smoking, physical activity and nutrition) will be excluded. Furthermore, studies will be excluded if information in the full-text article is insufficient (even after corresponding authors are contacted to obtain the required information). Reviews, commentaries, conference abstracts, editorials, and any other non-original research will also be excluded.

### Data sources and search strategy

#### Electronic searches

The following ten databases will be searched from the inception of the databases to March 2017 to identify potentially eligible studies: Cumulative Index to Nursing and Allied Health Literature (CINAHL), Cochrane Central Register of Controlled Trials (CENTRAL), Education Source, Excerpta Medica Database (EMBASE), Literatura Latino Americana e do Caribe em Ciências da Saúde (LILACS), Medical Literature Analysis and Retrieval System Online (MEDLINE), Occupational Therapy Systematic Evaluation of Evidence (OTSeeker), Physiotherapy Evidence Database (PEDro), PsycINFO, and SPORTDiscus. The search strategy was developed in collaboration with an experienced health sciences university librarian for MEDLINE and refined based on expert feedback from two members of the Knowledge Synthesis Group at the Ottawa Hospital Research Institute using the Peer Review of Electronic Search Strategies (PRESS) evidence-based checklist [39, 40]. The search strategy includes relevant Medical Subject Heading terms and keywords covering the target population and intervention, as well as a highly sensitive search filter for identifying controlled trials developed by the Cochrane Collaboration [41]. However, the latter was modified in an effort to maximize search strategy sensitivity while maintaining precision. The MEDLINE search strategy [see Additional file 2] was then translated for use in the nine other databases to be searched and peer reviewed by the two same members of the Knowledge Synthesis Group at the Ottawa Hospital Research Institute using the PRESS evidence-based checklist [39, 40].

#### Searching other resources

Reference lists from relevant studies retrieved during the databases searches will be hand searched to identify additional studies that may be missed during the initial search.

### Study selection

Studies identified during the database searches will be exported into a reference management software (i.e. EndNote; [42]), and duplicate records will be removed. Two reviewers will then independently review all titles and abstracts using the aforementioned inclusion/exclusion criteria to select articles for full-text review. Studies that do not meet inclusion/exclusion criteria based on both authors’ review will be discarded. Otherwise, the full-text article will be retrieved and assessed. When uncertainty or disagreement exists, the full-text article will be retrieved and assessed. Next, both reviewers will independently review the full-text of all potentially relevant studies using the first two pages of the data extraction form to establish eligibility [see Additional file 3]. The data extraction form was developed following the guidelines provided in the Cochrane Handbook for Systematic Reviews of Interventions [41]. Studies that do not meet the inclusion criteria will be excluded, and the reasons for exclusion will be recorded. While reviewing the full-text of all potentially relevant studies, both reviewers will also scan the reference lists to identify other potentially relevant studies. If any are identified, both reviewers will independently review the full-text(s) to determine eligibility. Any disagreements between the two reviewers as to the relevance of the study during the full-text review will be resolved through discussion and adjudication of a third reviewer. In accordance with the Preferred Reporting Items for Systematic Review and Meta-Analysis (PRISMA) statement [43, 44], a flow diagram will be prepared detailing the number of records scanned, included, and excluded at each stage of the process. The inter-rater agreement between both reviewers on eligibility will be computed, and the level of agreement will be reported.

## Data extraction

Data from all studies meeting inclusion criteria will be extracted by two reviewers independently using the data extraction form [see Additional file 3], which will be pilot tested with a representative sample of studies to identify any data that are missing from the form or that are nonessential. Should revisions be necessary after the pilot testing, these will be made prior to completing the remaining data extraction. The data extraction form has been developed to collect information covering: (1) general information (i.e. publication type, first author, funding source, potential conflict), (2) participant characteristics (i.e. age, sex, sample size, method of randomization), (3) intervention information (i.e. description of intervention groups), and (4) outcomes (i.e. primary and secondary outcomes, the time-points measured or reported). After completing the data extraction form independently, both reviewers will meet and compare their data extraction forms to ensure completeness and accuracy. Any disagreements will be resolved through discussion and adjudication of a third reviewer.

## Assessment of risk of bias of the included studies

Two reviewers will independently judge each study reviewed using the Cochrane Collaborations’ tool for assessing risk of bias [see Additional file 4] provided in the Cochrane Handbook for Systematic Reviews of Interventions [41]. This tool is comprised of six domains: (1) selection bias (two items; e.g. sequence generation, allocation concealment), (2) performance bias (one item; e.g. blinding of participants and personnel), (3) detection bias (one item; e.g. blinding of outcome assessment), (4) attrition bias (one item; e.g. incomplete outcome data), (5) reporting bias (one item; e.g. selective outcome reporting), and (6) other bias (one item; e.g. other sources of bias). Following recommendations [41], judgements of “high risk” for each domain will be given when there are systematic differences between: (1) baseline characteristics of the groups that are compared (i.e. selection bias), (2) groups in the care that is provided or in exposure to factors other than the interventions of interest (i.e. performance bias), (3) groups in how outcomes are determined (i.e. detection bias), (4) groups in withdrawals from a study (i.e. attrition bias), and/or (5) differences between reported and unreported findings (i.e. reporting bias). “Low risk” will be given when there are no systematic differences, whereas “unclear risk” will be given when there is a lack of information or uncertainty over the potential for risk of bias. Both reviewers will meet to compare their independent judgements, and any disagreement will be resolved through discussion and adjudication of a third reviewer. Judgement for each domain will be presented separately for each study in a “Risk of Bias in Included Studies” table [see Additional file 5].

## Data analysis

### Descriptive analysis

A summary of the results from the reviewed studies will be presented in “Characteristics of Included Studies” and “Summary of Findings” tables using the information gathered from the data extraction form. In addition, a narrative synthesis of the results from the reviewed studies will also be prepared. The tables and the narrative synthesis will follow the Guidance on the Conduct of Narrative Synthesis in Systematic Reviews [45] and the Cochrane Handbook for Systematic Reviews of Interventions [41].

### Statistical analysis

A member from the Knowledge Synthesis Group at Ottawa Hospital Research Institute will perform quantitative analyses to provide summaries of intervention effects for each study by calculating risk ratios (for dichotomous outcomes) or standardized mean differences (for continuous outcomes) using the data presented in the full-text articles or obtained from the corresponding author(s). Additionally, corresponding 95% confidence intervals (CI) for each assessed outcome will be calculated and interpreted based on the recommendations outlined in the Cochrane Handbook for Systematic Reviews of Interventions (i.e. null effect if CI crosses over 0, small effect if CI is wide, and large effect if CI is narrow; [41]). Next, if there are a sufficient number of studies in which the same outcome(s) was/were measured, individual effect sizes will be pooled using fixed effects methods, which assume there is a common underlying effect and the variability observed between studies is attributed to chance alone. Random effects methods, which take into account between-study heterogeneity, will also be used for comparison. Heterogeneity between the findings of the reviewed studies will be assessed using the *I* ^2^ statistic. An *I* ^2^ value greater than 50% will be considered indicative of substantial heterogeneity. Of note, in the absence of heterogeneity, fixed effects and random effects methods yield the same results. Sensitivity analyses will be conducted to assess the effect of removing studies with a “high risk” of bias, as is recommended by Liberati et al. [43]. Statistical analyses will be performed with SAS software (SAS Institute Inc., Cary, NC, USA).

## Discussion

Promoting participation in physical activity among cancer survivors may help to mitigate the negative side effects commonly reported during and post-treatment such as musculoskeletal limitations, impaired cardiovascular system functioning, cancer-related fatigue, cognitive problems, anxiety, and depression [12–18]. Whereas health care providers are in a unique position to influence cancer survivors’ health behaviours, including their participation in physical activity [22–24], few recommend it [27–30]. One reason physical activity recommendations have not been integrated into practice is that health care providers may doubt their ability to impact their patients’ participation in physical activity [29, 30, 33]. This systematic review and meta-analysis will provide insight into the effectiveness of health care provider physical activity recommendations on participation in physical activity among cancer survivors, a population who has a lot to gain from being active [12–18].

The results will be of interest for a broad audience, including health care or allied health care providers (e.g. oncologists, nurses, physicians, physiotherapists, kinesiologists), guideline developers, policy makers, and researchers. Indeed, it is hoped that this review will provide evidence to reduce the uncertainty about the effectiveness of incorporating physical activity recommendations into standard care in an effort to ensure physical activity recommendations are integrated into regular practice, and thus reduce the gap between research, practice, and policy. Furthermore, it is hoped that the identification of knowledge gaps will encourage further research in this area.

## Additional files

Additional file 1: PRISMA-P 2015 checklist. (PDF 159 kb)

Additional file 2: MEDLINE search strategy. (PDF 59 kb)

Additional file 3: Data extraction form. (PDF 129 kb)

Additional file 4: The Cochrane Collaboration’s tool for assessing risk of bias. (PDF 68 kb)

Additional file 5: Sample risk of bias figure. (JPG 82 kb)

## Abbreviations

CENTRAL: Cochrane Central Register of Controlled Trials
CI: Confidence interval
CINAHL: Cumulative Index of Nursing and Allied Health Literature
EMBASE: Excerpta Medica Database
LILACS: Literatura Latinoamericana y del Caribe en Ciencias de la Salud
MEDLINE: Medical Literature Analysis and Retrieval System Online
OTSeeker: Occupational Therapy Systematic Evaluation of Evidence
PEDro: Physiotherapy Evidence Database
PRESS: Peer Review of Electronic Search Strategies
PRISMA: Preferred Reporting Items for Systematic Review and Meta-Analyses
PRISMA-P: Preferred Reporting Items for Systematic Review and Meta-Analysis Protocols

## References

[1] American Cancer Society (2014). Cancer facts & figures 2014.

[2] Canadian Cancer Society’s Advisory Committee on Cancer Statistics (2015). Canadian cancer statistics 2015.

[3] Alfano CM, Rowland JH (2006). Recovery issues in cancer survivorship: a new challenge for supportive care. Cancer J.

[4] de Boer AG, Taskila T, Ojajärvi A, van Dijk FJ, Verbeek JH (2009). Cancer survivors and unemployment: a meta-analysis and meta-regression. JAMA.

[5] Fossa SD, Vassilopoulou-Sellin R, Dahl AA (2008). Long term physical sequelae after adult-onset cancer. J Cancer Surviv.

[6] Stein KD, Syrjala KL, Andrykowski MA (2008). Physical and psychological long-term and late effects of cancer. Cancer.

[7] Taskila T, Lindbohm ML (2007). Factors affecting cancer survivors’ employment and work ability. Acta Oncol.

[8] Bodai BI, Tuso P (2015). Breast cancer survivorship: a comprehensive review of long-term medical issues and lifestyle recommendations. Perm J.

[9] Nord C, Mykletun A, Thorsen L, Bjøro T, Fosså SD (2005). Self-reported health and use of health care services in long-term cancer survivors. Int J Cancer.

[10] Pollack LA, Adamache W, Ryerson AB, Eheman CR, Richardson LC (2009). Care of long-term cancer survivors: physicians seen by Medicare enrollees surviving longer than 5 years. Cancer.

[11] Simard S, Thewes B, Humphris G, Dixon M, Hayden C, Mireskandari S, Ozakinci G (2013). Fear of cancer recurrence in adult cancer survivors: a systematic review of quantitative studies. J Cancer Surviv.

[12] Ballard-Barbash R, Friedenreich CM, Courneya KS, Siddiqi SM, McTiernan A, Alfano CM (2012). Physical activity, biomarkers, and disease outcomes in cancer survivors: a systematic review. J Natl Cancer Inst.

[13] Mishra SI, Scherer RW, Geigle PM, Berlanstein DR, Topaloglu O, Gotay CC, Snyder C (2012). Exercise interventions on health-related quality of life for cancer survivors. Cochrane Database Syst Rev.

[14] Mishra SI, Scherer RW, Snyder C, Geigle PM, Berlanstein DR, Topaloglu O (2012). Exercise interventions on health-related quality of life for people with cancer during active treatment. Cochrane Database Syst Rev.

[15] Sabiston CM, Brunet J (2011). Reviewing the benefits of physical activity during cancer survivorship. American J Lifestyle Med.

[16] Speck RM, Courneya KS, Mâsse LC, Duval S, Schmitz KH (2010). An update of controlled physical activity trials in cancer survivors: a systematic review and meta-analysis. J Cancer Surviv.

[17] Schmid D, Leitzmann MF (2014). Association between physical activity and mortality among breast cancer and colorectal cancer survivors: a systematic review and meta-analysis. Ann Oncol.

[18] Barbaric M, Brooks E, Moore L, Cheifetz O (2010). Effects of physical activity on cancer survival: a systematic review. Physiother Can.

[19] Mason C, Alfano CM, Smith AW, Wang CY, Neuhouser ML, Duggan C, Bernstein L, Baumgartner KB, Baumgartner RN, Ballard-Barbash R, McTiernan A (2013). Long-term physical activity trends in breast cancer survivors. Cancer Epidemiol Biomarkers Prev.

[20] Schmitz KH, Courneya KS, Matthews C, Demark-Wahnefried W, Galvão DA, Pinto BM, Irwin ML, Wolin KY, Segal RJ, Lucia A, Schneider CM, von Gruenigen VE, Schwartz AL (2010). American College of Sports Medicine. American College of Sports Medicine roundtable on exercise guidelines for cancer survivors. Med Sci Sports Exerc.

[21] Demark-Wahnefried W, Aziz NM, Rowland JH, Pinto BM (2005). Riding the crest of the teachable moment: promoting long-term health after the diagnosis of cancer. J Clin Oncol.

[22] Orrow G, Sanderson S, Sutton S (2012). Effectiveness of physical activity promotion based in primary care: systematic review and meta-analysis of randomised controlled trials. BMJ.

[23] Sorensen JB, Skovgaard T, Puggaard L (2006). Exercise on prescription in general practice: a systematic review. Scand J Prim Health Care.

[24] Vuori IM, Lavie CJ, Blair SN (2013). Physical activity promotion in the health care system. Mayo Clin Proc.

[25] Eakin EG, Glasgow RE, Riley KM (2000). Review of primary care-based physical activity intervention studies: effectiveness and implications for practice and future research. J Fam Pract.

[26] Swinburn BA, Walter LG, Arroll B, Tilyard MW, Russell DG (1998). The green prescription study: a randomized controlled trial of written exercise advice provided by general practitioners. Am J Public Health.

[27] Daley AJ, Bowden SJ, Rea DW, Billingham L, Carmicheal AR (2008). What advice are oncologists and surgeons in the United Kingdom giving to breast cancer patients about physical activity?. Int J Behav Nutr Phys Act.

[28] Demark-Wahnefried W, Peterson B, McBride C, Lipkus I, Clipp E (2000). Current health behaviors and readiness to pursue life-style changes among men and women diagnosed with early stage prostate and breast carcinomas. Cancer.

[29] Jones LW, Courneya KS, Peddle C, Mackey JR (2005). Oncologists’ opinions towards recommending exercise to patients with cancer: a Canadian national survey. Support Care Cancer.

[30] Spellman C, Craike M, Livingston P (2014). Knowledge, attitudes and practices of clinicians in promoting physical activity to prostate cancer survivors. Health Educ J.

[31] Karvinen KH, McGourty S, Parent T, Walker PR (2012). Physical activity promotion among oncology nurses. Cancer Nurs.

[32] Williams K, Beeken RJ, Fisher A, Wardle J (2015). Health professionals’ provision of lifestyle advice in the oncology context in the United Kingdom. Eur J Cancer Care (Engl).

[33] Hebert ET, Caughy MO, Shuval K (2012). Primary care providers’ perceptions of physical activity counselling in a clinical setting: a systematic review. Br J Sports Med.

[34] Moher D, Shamseer L, Clarke M, Ghersi D, Liberati A, Petticrew M, Shekelle P, Stewart LA, the PRISMA-P Group (2015). Preferred reporting items for systematic review and meta-analysis protocols (PRISMA-P) 2015 statement. Syst Rev.

[35] Shamseer L, Moher D, Clarke M, Ghersi D, Liberati A, Petticrew M, Shekelle P, Stewart LA, the PRISMA-P Group (2016). Preferred reporting items for systematic review and meta-analysis protocols (PRISMA-P) 2015: elaboration and explanation. BMJ.

[36] National Cancer Institute. National Cancer Institute dictionary of cancer terms: survivor. http://www.cancer.gov/publications/dictionaries/cancer-terms?search=survivor. Accessed 20 Aug 2016.

[37] Sheikh A, Smeeth L, Ashcroft R (2002). Randomised controlled trials in primary care: scope and application. Br J Gen Pract.

[38] Sibbald B, Roland M (1998). Understanding controlled trials. Why are randomised controlled trials important?. BMJ.

[39] McGowan J, Sampson M, Salzwedel DM, Cogo E, Foerster V, Lefebvre C (2016). PRESS Peer Review of Electronic Search Strategies: 2015 guideline explanation and elaboration (PRESS E&E).

[40] McGowan J, Sampson M, Salzwedel DM, Cogo E, Foerster V, Lefebvre C (2016). PRESS Peer Review of Electronic Search Strategies: 2015 guideline statement. J Clin Epidemiol.

[41] Higgins JPT, Green S. Cochrane handbook for systematic reviews of interventions. Version 5.1.0. http://www.cochrane-handbook.org. Accessed 20 Aug 2016.

[42] Thomas Reuters. EndNote X7.4. 1988–2015. Software.

[43] Liberati A, Altman DG, Tetzlaff J, Mulrow C, Gøtzsche PC, Ioannidis JPA, Clarke M, Devereaux PJ, Kleijnen J, Moher D (2009). The PRISMA statement for reporting systematic reviews and meta-analyses of studies that evaluate health care interventions: explanation and elaboration. J Clin Epidemiol.

[44] Moher D, Liberati A, Tetzlaff J, Altman DG, the PRISMA Group (2009). Preferred Reporting Items for Systematic Reviews and Meta-Analyses Preferred reporting items for systematic reviews and meta-analyses: the PRISMA statement. PLoS Med.

[45] Popay J, Roberts H, Sowden A, Petticrew M, Arai L, Rodgers M. Guidance on the conduct of narrative synthesis in systematic reviews. 2005. http://www.lancs.ac.uk/shm/research/nssr/index.htm. Accessed 21 Aug 2016.

