# Supplementary material for: The effectiveness of health care provider physical activity recommendations in cancer survivors: a systematic review and meta-analysis protocol
Source: Syst Rev. 2017 Mar 27;6:66. doi: 10.1186/s13643-017-0453-3 (PMC5369014; doi:10.1186/s13643-017-0453-3)
Supplement: Supplementary file 1 — PRISMA-P 2015 checklist. (PDF 159 kb) [file 13643_2017_453_MOESM1_ESM.pdf]

Additional file 1: PRISMA-P 2015 checklist

This checklist has been adapted for use with systematic review protocol submissions to BioMed Central journals from Table 3 in Moher D, et al. Preferred reporting items for systematic review and meta-analysis protocols (PRISMA-P) 2015 statement. Systematic Reviews. 2015:4:1.

An Editorial from the Editors-in-Chief of *Systematic Reviews* details why this checklist was adapted - Moher D, Stewart L, Shekelle P. Implementing PRISMA-P: recommendations for prospective authors. Systematic Reviews. 2016:5:15.

Section/topic	#	Checklist item	Information reported		Page number(s), section, line number(s)*
			Yes	No	
**ADMINISTRATIVE INFORMATION**					
**Title**					
Identification	1a	Identify the report as a protocol of a systematic review	☒	☐	Page 1, title section, lines 1-5<br>Page 2, methods section, lines 51-58
Update	1b	If the protocol is for an update of a previous systematic review, identify as such	☐	☒	This protocol is not an update of a previous systematic review
**Registration**	2	If registered, provide the name of the registry (e.g., PROSPERO) and registration number in the Abstract	☒	☐	Once this protocol has undergone peer review and has been accepted for publication, the published review protocol will be registered with PROSPERO
**Authors**					
Contact	3a	Provide name, institutional affiliation, and e-mail address of all protocol authors; provide physical mailing address of corresponding author	☒	☐	Page 1, author section, lines 6-7<br>Page 1, brief author details section, lines 34-39<br>Page 5, author details section, lines 52-63

BioMed Central
The Open Access Publisher

Section/topic	#	Checklist item	Information reported Yes	Information reported No	Page number(s), section, line number(s)*
Contributions	3b	Describe contributions of protocol authors and identify the guarantor of the review	☒	☐	Page 5, authors' contributions section, lines 34-37
**Amendments**	4	If the protocol represents an amendment of a previously completed or published protocol, identify as such and list changes; otherwise, state plan for documenting important protocol amendments	☐	☒	This protocol does not represent an amendment of a previously completed or published protocol
**Support**					
Sources	5a	Indicate sources of financial or other support for the review	☒	☐	Page 5, funding section, lines 33-36
Sponsor	5b	Provide name for the review funder and/or sponsor	☒	☐	Page 5, funding section, lines 33-36
Role of sponsor/funder	5c	Describe roles of funder(s), sponsor(s), and/or institution(s), if any, in developing the protocol	☒	☐	Page 5, funding section, lines 33-36
**INTRODUCTION**					
**Rationale**	6	Describe the rationale for the review in the context of what is already known	☒	☐	Page 1, background section, lines 27-51<br>Page 2, background section, lines 1-50
**Objectives**	7	Provide an explicit statement of the question(s) the review will address with reference to participants, interventions, comparators, and outcomes (PICO)	☒	☐	Page 2, background section, lines 46
**METHODS**					
**Eligibility criteria**	8	Specify the study characteristics (e.g., PICO, study design, setting, time frame) and report characteristics (e.g., years considered, language, publication status) to be used as criteria for eligibility for the review	☒	☐	Page 2, inclusion/exclusion section, lines 59-103<br>Page 3, inclusion/exclusion section, lines 1-22
**Information sources**	9	Describe all intended information sources (e.g., electronic databases, contact with study authors, trial registers, or other grey literature sources) with planned dates of coverage	☒	☐	Page 3, data sources and search strategy section, lines 23-58

Section/topic	#	Checklist item	Information reported		Page number(s), section, line number(s)*
			Yes	No	
**Search strategy**	10	Present draft of search strategy to be used for at least one electronic database, including planned limits, such that it could be repeated	☒	☐	Page 3, data sources and search strategy section, line 49<br>See Additional file 2
STUDY RECORDS					
Data management	11a	Describe the mechanism(s) that will be used to manage records and data throughout the review	☒	☐	Page 3, study selection section, lines 60-63
Selection process	11b	State the process that will be used for selecting studies (e.g., two independent reviewers) through each phase of the review (i.e., screening, eligibility, and inclusion in meta-analysis)	☒	☐	Page 3, study selection section, lines 59-92
Data collection process	11c	Describe planned method of extracting data from reports (e.g., piloting forms, done independently, in duplicate), any processes for obtaining and confirming data from investigators	☒	☐	Page 3, data extraction section, lines 93-103<br>Page 4, data extraction section, lines 1-10
**Data items**	12	List and define all variables for which data will be sought (e.g., PICO items, funding sources), any pre-planned data assumptions and simplifications	☒	☐	Page 3, data extraction section, lines 101-103<br>Page 4, data extraction section, lines 1-6
**Outcomes and prioritization**	13	List and define all outcomes for which data will be sought, including prioritization of main and additional outcomes, with rationale	☒	☐	Page 3, types of outcome measures section, lines 7-13
**Risk of bias in individual studies**	14	Describe anticipated methods for assessing risk of bias of individual studies, including whether this will be done at the outcome or study level, or both; state how this information will be used in data synthesis	☒	☐	Page 4, assessment of risk of bias of the included studies section, lines 11-42

Section/topic	#	Checklist item	Information reported		Page number(s), section, line number(s)*
			Yes	Not	
DATA					
**Synthesis**	15a	Describe criteria under which study data will be quantitatively synthesized	☒	☐	Page 4, statistical analysis section, lines 55-85
	15b	If data are appropriate for quantitative synthesis, describe planned summary measures, methods of handling data, and methods of combining data from studies, including any planned exploration of consistency (e.g., I^2, Kendall's tau)	☒	☐	Page 4, statistical analysis section, lines 55-85
	15c	Describe any proposed additional analyses (e.g., sensitivity or subgroup analyses, meta-regression)	☒	☐	Page 4, statistical analysis section, lines 55-85
	15d	If quantitative synthesis is not appropriate, describe the type of summary planned	☒	☐	Page 2, types of studies section, 100-103<br>Page 3, types of studies section, 1-6<br>Page 4, statistical analysis section, lines 55-85
**Meta-bias(es)**	16	Specify any planned assessment of meta-bias(es) (e.g., publication bias across studies, selective reporting within studies)	☐	☒	This has not been specified
**Confidence in cumulative evidence**	17	Describe how the strength of the body of evidence will be assessed (e.g., GRADE)	☐	☒	This has not been described

* Line numbers re-start on the first line of every page and do not include spaces.
