# Supplementary material for: The effectiveness of health care provider physical activity recommendations in cancer survivors: a systematic review and meta-analysis protocol
Source: Syst Rev. 2017 Mar 27;6:66. doi: 10.1186/s13643-017-0453-3 (PMC5369014; doi:10.1186/s13643-017-0453-3)
Supplement: Supplementary file 2 — MEDLINE search strategy. (PDF 59 kb) [file 13643_2017_453_MOESM2_ESM.pdf]

Additional file 2: MEDLINE search strategy

1. exp exercise/
2. physical fitness/
3. motor activity/
4. exercis*.tw,kw.
5. (physical* adj2 (activ* or train* or exercis* or fit*4)).tw,kw.
6. (physical* adj2 condition*).tw,kw.
7. aerobic.tw,kw.
8. endurance*.tw,kw.
9. (strength adj1 (exercis* or train*)).tw,kw.
10. flexibility.tw,kw.
11. (resistance adj1 (exercis* or train*)).tw,kw.
12. ((muscl* or muscul*) adj2 (exercis* or train*)).tw,kw.
13. stretching.tw,kw.
14. (circuit-based adj1 exercis*).tw,kw.
15. ((cool-down or cooldown) adj1 exercis*).tw,kw.
16. ((muscl* adj1 stretch*) or (motion adj1 therap*)).tw,kw.
17. (plyometric adj1 exercis*).tw,kw.
18. (swimming or swim).tw,kw.
19. swimming/ or running/ or walking/
20. (running or run).tw,kw.
21. (walking or walk).tw,kw.
22. ((warm-up or warmup) adj1 exercis*).tw,kw.
23. or/1-22
24. counseling/
25. directive counseling/
26. motivational interviewing/
27. patient education as topic/
28. prescriptions/
29. (prescript* or prescrib*).tw,kw.
30. (advis* or advice).tw,kw.
31. counsel*.tw,kw.
32. (motivational adj1 interview*).tw,kw.
33. (educat* adj3 health*).tw,kw.
34. (patient* adj2 educat*).tw,kw.
35. health promotion/
36. (health* adj3 promot*).tw,kw.
37. "referral and consultation"/
38. patient education handout/
39. or/24-38
40. 23 and 39
41. exp exercise therapy/
42. (exercis* adj3 (promot* or recommend* or refer or refers or referred or referral* or therap* or counsel* or prescrip* or prescrib* or rehab* or coach*)).tw,kw.

43. (physical* adj2 (activ* or train* or fit*4) adj3 (promot* or recommend* or refer or refers or referred or referral* or therap* or counsel* or prescrip* or prescrib* or rehab* or coach*)).tw,kw.
44. (kinesiotherap* or kinesitherap* or kinesiatric*).tw,kw.
45. or/40-44
46. exp neoplasms/
47. (cancer* or tumo?r* or oncolog*).tw,kw.
48. (leuk?emia* or carcinoma* or adeno-carcinoma* or neoplas* or lymphoma* or malignan* or melanoma* or metasta* or sarcoma* or adenoma* or adenocarcinoma* or blastoma* or mesothelioma*).tw,kw.
49. or/46-48
50. 45 and 49
51. randomized controlled trial.pt.
52. controlled clinical trial.pt.
53. randomi?ed.tw,kw.
54. placebo*.tw,kw.
55. exp clinical trials as topic/
56. randomly.tw,kw.
57. trial.ti.
58. double-blind method/
59. single blind method/
60. ((singl* or doubl* or tripl* or treb*) adj (blind*3 or mask*3)).tw,kw.
61. placebos/
62. (nRCT$1 or RCT or RCTs).tw,kw.
63. (nonrandom* or non-random* or quasi-random* or quasi-experiment* or (quasi adj1 experiment*)).tw,kw.
64. historically controlled study/ or controlled before-after studies/ or case-control studies/
65. interrupted time series analysis/
66. (historically adj2 controlled).tw,kw.
67. (time adj1 series).tw,kw.
68. case-control*.tw,kw.
69. (case adj1 control*).tw,kw.
70. (controlled adj3 study).tw,kw.
71. or/51-70
72. 50 and 71
73. exp animals/ not humans.sh.
74. 72 not 73
75. exp infant/ not exp adults/
76. exp child/ not exp adults/
77. adolescent/ not exp adults/
78. or/75-77
79. 74 not 78
80. editorial.pt.
81. news.pt.
82. comment.pt.
83. interview.pt.

84. or/80-83
85. 79 not 84
