# Supplementary material for: The effectiveness of health care provider physical activity recommendations in cancer survivors: a systematic review and meta-analysis protocol
Source: Syst Rev. 2017 Mar 27;6:66. doi: 10.1186/s13643-017-0453-3 (PMC5369014; doi:10.1186/s13643-017-0453-3)
Supplement: Supplementary file 3 — Data extraction form. (PDF 129 kb) [file 13643_2017_453_MOESM3_ESM.pdf]

**Additional file 3: Data extraction form**

| Study identification: | Date form completed: | |
|---|---|---|
| First author: | Year of study: | Name of extractor: |
| Full citation (including doi): | | |

## GENERAL INFORMATION

| | |
|---|---|
| Publication type: ☐ Journal article ☐ Abstract ☐ Other (specify: ________________) | |
| Country of study: | |
| Funding source of study: | |
| Potential conflict of interest from funding source? | ☐ Yes ☐ No ☐ Unclear |

## STUDY ELIGIBILITY

| **Study characteristics** | | | **Page/ Para/ Fig #** |
|---|---|---|---|
| Type of study | ☐ Randomized controlled trial<br>☐ Controlled clinical trial<br>☐ Other (specify: __________________) | | |
| | Does the study design meet the criteria for inclusion? | ☐ Yes ☐ No →**Exclude** ☐ Unclear | |
| | Description in text: | | |
| Participants | Describe the participants: | | |
| | Do the participants meet the criteria for inclusion? | ☐ Yes ☐ No →**Exclude** ☐ Unclear | |
| | Description in text: | | |

**STUDY ELIGIBILTY (CONTINUED)**

| | | | |
|---|---|---|---|
| Type(s) of intervention(s) | Describe the intervention(s): | | |
| | Does/do the intervention(s) meet the criteria for inclusion? | ☐ Yes ☐ No →**Exclude** ☐ Unclear | |
| | Description in text: | | |
| Type(s) of outcome(s) assessed | Describe the physical activity outcome(s) assessed: | | |
| | Does/do the physical activity outcome(s) meet the criteria for inclusion? | ☐ Yes ☐ No →**Exclude** ☐ Unclear | |
| | Description in text: | | |
| | Were additional outcomes assessed? | ☐ Yes ☐ No →**Exclude** ☐ Unclear | |
| | If yes, what additional outcome(s) was/were assessed? | | |
| | Description in text: | | |

**SUMMARY OF ASSESSMENT FOR INCLUSION**

| | |
|---|---|
| ☐ Include in review ☐ Exclude from review ☐ Unclear | |
| Independently assessed and then compared?<br>☐ Yes ☐ No | Disagreement resolved?<br>☐ Yes ☐ No |
| Request further details?<br>☐ Yes ☐ No | Contact details of corresponding authors: |
| Notes: | |

**DO NOT PROCEED IF PAPER EXCLUDED FROM REVIEW**

**STUDY DETAILS**

| **Study intention** | **Descriptions as stated in the report/paper** | **Page/ Para/ Fig #** |
|---|---|---|
| Aim of intervention(s) | What was the problem that the intervention(s) was/were designed to address? | |
| Aim(s) of study | What was the study designed to assess? Is/are the aim clearly stated? | |
| Total study duration | | |
| Notes: | | |

| **Methods** | **Descriptions as stated in the report/paper** | **Page/ Para/ Fig #** |
|---|---|---|
| Method(s) of recruitment of participants *(How were potential participants approached and invited to participate? Where were participants recruited from? Does this differ from the intervention setting?)* | | |
| Inclusion/exclusion criteria for participation in study | Inclusion:<br><br>Exclusion: | |
| Representativeness of sample:<br>Are participants in the study likely to be representative of the target population? | ☐ Yes ☐ No ☐ Unclear<br>Details: | |
| Total number of intervention groups | | |
| Sample size calculation:<br>What assumptions were made?<br>Were these assumptions appropriate? | Assumptions:<br>☐ Yes ☐ No ☐ Unclear | |
| What was the unit of randomization (if applicable)?<br>Was allocation by individuals or cluster/groups? | Unit:<br><br>☐ Yes ☐ No ☐ Unclear | |
| What was the unit of analysis?<br>Is this the same as the unit of randomization? | Unit:<br>☐ Yes ☐ No ☐ Unclear | |
| Statistical method(s) used and appropriateness of these methods | | |
| Notes: | | |

| **Participants** | **Include information for each group (i.e., intervention(s) and control(s)) under study** | **Page/ Para/ Fig #** |
|---|---|---|
| What percentage of selected individuals agreed to participate? | | |
| Total number randomized (or total population at start of study for controlled clinical trials) | | |
| Number of individuals allocated to each group | | |
| Where there any significant baseline imbalances? | ☐ Yes ☐ No ☐ Unclear<br>Details: | |
| Number and reason for withdrawals and exclusions for each group | | |
| What percentage of participants completed the study? | | |
| What percentage of participants received the allocated intervention(s)? | | |
| Is the analysis performed by intervention(s) allocation status (intention-to-treat) rather than the actual intervention received?<br>Have any attempts been made to impute missing data? If imputation was used, what imputation method(s) was used? | | |
| Age (median, mean, range, and variance, if possible) | Median: Mean: Range:<br>Standard deviation (or other variance): | |
| Sex | ☐ Male (n= ) ☐ Female (n= ) | |
| Race/Ethnicity | | |
| Principal health problem (include type of cancer, stage of disease, and treatment status if possible) | | |

| | | |
|---|---|---|
| Comorbid health condition(s)? | | |
| Notes: | | |

| **Intervention group 1** *(copy and paste table if multiple intervention groups)* | | **Page/ Para/ Fig #** |
|---|---|---|
| Specify setting (e.g., multi-centre, community clinic, GP clinic) | | |
| Theoretical framework | ☐ Yes ☐ No ☐ Unclear<br>Describe (provide key references): | |
| Content (list the strategies intended and delivered) | Details: | |
| Delivery (e.g., timing, frequency, duration, intensity, fidelity) | | |
| Providers (e.g., who, number, profession, training) | | |
| Follow-up assessments? | ☐ Yes ☐ No ☐ Unclear<br>If yes, duration: | |
| What are the moderators/mediators of changes stated in the study? | | |
| Control/comparison (what information is provided about what the control or comparison group received?) | Details: | |
| Notes: | | |

**OUTCOMES**

| | **Outcome 1** | **Page/ Para/ Fig #** |
|---|---|---|
| Outcome name and definition | | |
| Time points measured | | |
| Time points reported | | |
| How is the measure applied? (e.g., telephone survey, mail survey, in person, routinely collected data) | | |
| How is the outcome assessed? | ☐ Self-reported ☐ Objective<br>Describe: | |
| Unit of measurement (if relevant) | | |
| Has the outcome measure validated? | ☐ Yes ☐ No ☐ Unclear | |
| Is the outcome measure reliable? | ☐ Yes ☐ No ☐ Unclear | |
| Notes: | | |

| *(copy and paste table if multiple intervention groups)* | **Outcome 2….k** | **Page/ Para/ Fig #** |
|---|---|---|
| Outcome name and definition | | |
| Time points measured | | |
| Time points reported | | |
| How is the measure applied? (e.g., telephone survey, mail survey, in person, routinely collected data) | | |
| How is the outcome assessed? | ☐ Self-reported ☐ Objective<br>Describe: | |
| Unit of measurement (if relevant) | | |
| Has the outcome measure validated? | ☐ Yes ☐ No ☐ Unclear | |
| Is the outcome measure reliable? | ☐ Yes ☐ No ☐ Unclear | |
| Notes: | | |

**RESULTS**

<table>
<tr><th></th><th colspan="6">For continuous outcomes</th><th>Page/ Para/ Fig #</th></tr>
<tr><td>Description (as reported in text)</td><td colspan="6"></td><td></td></tr>
<tr><td>Comparison</td><td colspan="6"></td><td></td></tr>
<tr><td>Outcome(s)</td><td colspan="6"></td><td></td></tr>
<tr><td>Subgroups</td><td colspan="6"></td><td></td></tr>
<tr><td>Time points</td><td colspan="6"></td><td></td></tr>
<tr><td>Post-intervention or change from baseline?</td><td colspan="6"></td><td></td></tr>
<tr><td rowspan="3">Results</td><td colspan="3">Intervention</td><td colspan="3">Comparison</td><td></td></tr>
<tr><td>Mean/ Median</td><td>SD (or other variance)</td><td>Number of participants</td><td>Mean/ Median</td><td>SD (or other variance)</td><td>Number of participants</td><td rowspan="2"></td></tr>
<tr><td></td><td></td><td></td><td></td><td></td><td></td></tr>
<tr><td>Any other results reported</td><td colspan="6"></td><td></td></tr>
<tr><td>Number of missing participants and reasons</td><td colspan="6"></td><td></td></tr>
<tr><td>Re-analysis required?</td><td colspan="6">☐ Yes ☐ No ☐ Unclear<br>Reasons:</td><td></td></tr>
<tr><td>Re-analysis possible?</td><td colspan="6">☐ Yes ☐ No ☐ Unclear<br>Reasons:</td><td></td></tr>
<tr><td>Re-analyzed results</td><td colspan="6"></td><td></td></tr>
<tr><td colspan="8">Notes:</td></tr>
</table>

| | **For dichotomous or categorical outcomes** | | | | | | **Page/ Para/ Fig #** |
|---|---|---|---|---|---|---|---|
| Description (as reported in text) | | | | | | | |
| Comparison | | | | | | | |
| Outcome(s) | | | | | | | |
| Subgroups | | | | | | | |
| Time points | | | | | | | |
| Post-intervention or change from baseline? | | | | | | | |
| Results | Intervention | | | Comparison | | | |
| | Effect estimate | CI (or other variance) | Number of participants | Effect estimate | CI (or other variance) | Number of participants | |
| | | | | | | | |
| Any other results reported | | | | | | | |
| Number of missing participants and reasons | | | | | | | |
| Re-analysis required? | ☐ Yes ☐ No ☐ Unclear<br>Reasons: | | | | | | |
| Re-analysis possible? | ☐ Yes ☐ No ☐ Unclear<br>Reasons: | | | | | | |
| Re-analyzed results | | | | | | | |
| Notes: | | | | | | | |

**OTHER RELEVANT INFORMATION**

| | |
|---|---|
| Were outcomes relating to harms/adverse events of the intervention described? Include any data for these in the outcomes tables above | |
| Key conclusions of the study authors | |
| Could the inclusion of this study potentially bias the generalizability of the review? | |

**ATTACH CHECKLIST FOR ASSESSING RISK OF BIAS**
