# Supplementary material for: The effectiveness of health care provider physical activity recommendations in cancer survivors: a systematic review and meta-analysis protocol
Source: Syst Rev. 2017 Mar 27;6:66. doi: 10.1186/s13643-017-0453-3 (PMC5369014; doi:10.1186/s13643-017-0453-3)
Supplement: Supplementary file 4 — The Cochrane Collaboration’s tool for assessing risk of bias. (PDF 68 kb) [file 13643_2017_453_MOESM4_ESM.pdf]

Additional file 4: The Cochrane Collaboration's tool for assessing risk of bias

Domain	Support for judgment	Review authors' judgment
**Selection bias**		
Random sequence generation	Describe the method used to generate the allocation sequence in sufficient detail to allow an assessment of whether it should produce comparable groups.	Selection bias (biased allocation to interventions) due to inadequate generation of a randomized sequence.
Allocation concealment	Describe the method used to conceal the allocation sequence in sufficient detail to determine whether intervention allocations could have been foreseen in advance of, or during, enrolment.	Describe the method used to conceal the allocation sequence in sufficient detail to determine whether intervention allocations could have been foreseen in advance of, or during, enrolment.
**Performance bias**		
Blinding of participants and personnel<br>*Assessments should be made for each main outcome (or class of outcomes)*	Describe all measures used, if any, to blind study participants and personnel from knowledge of which intervention a participant received. Provide any information relating to whether the intended blinding was effective.	Performance bias due to knowledge of the allocated interventions by participants and personnel during the study.
**Detection bias**		
Blinding of outcome assessment<br>*Assessments should be made for each main outcome (or class of outcomes)*	Describe all measures used, if any, to blind outcome assessors from knowledge of which intervention a participant received. Provide any information relating to whether the intended blinding was effective.	Detection bias due to knowledge of the allocated interventions by outcome assessors.
**Attrition bias**		
Incomplete outcome data<br>*Assessments should be made for each main outcome (or class of outcomes)*	Describe the completeness of outcome data for each main outcome, including attrition and exclusions from the analysis. State whether attrition and exclusions were reported, the numbers in each intervention group (compared with total randomized	Attrition bias due to amount, nature or handling of incomplete outcome data.

	participants), reasons for attrition/exclusions where reported, and any re-inclusions in analyses performed by the review authors.	
**Reporting bias**		
Selective reporting	State how the possibility of selective outcome reporting was examined by the review authors, and what was found.	Reporting bias due to selective outcome reporting.
**Other bias**		
Other sources of bias	State any important concerns about bias not addressed in the other domains in the tool.<br><br>If particular questions/entries were pre-specified in the review's protocol, responses should be provided for each question/entry.	Bias due to problems not covered elsewhere in the table.
