# Supplementary figures and images for: The effectiveness of health care provider physical activity recommendations in cancer survivors: a systematic review and meta-analysis protocol
Source: Syst Rev. 2017 Mar 27;6:66. doi: 10.1186/s13643-017-0453-3 (PMC5369014; doi:10.1186/s13643-017-0453-3)

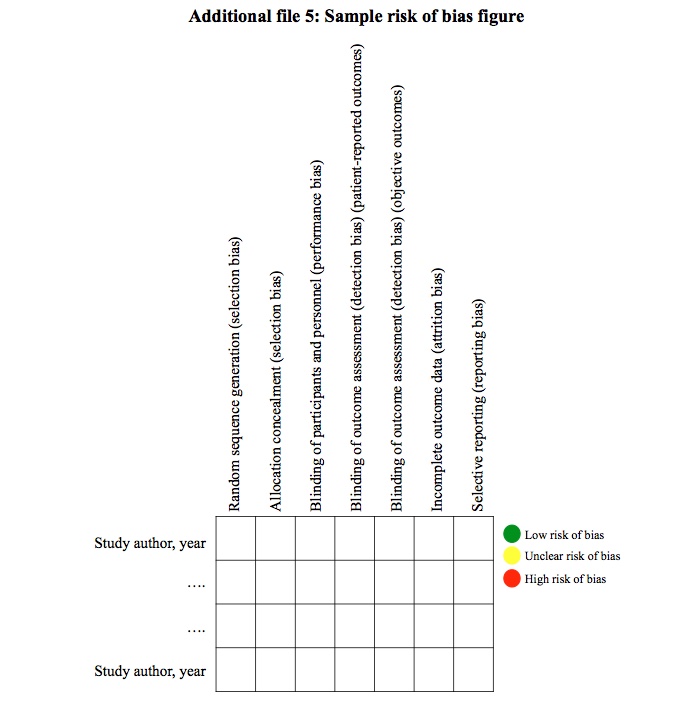

Supplement: Supplementary file 5 — Sample risk of bias figure. (JPG 82 kb) [file 13643_2017_453_MOESM5_ESM.jpg]
